# Thyroid Function and Metabolic Syndrome in Children and Adolescents with Neuromotor Disability

**DOI:** 10.3390/children9101531

**Published:** 2022-10-06

**Authors:** Valeria Calcaterra, Giacomo Biganzoli, Simona Ferraro, Alessandra Mari, Anna Mandelli, Valentina Fabiano, Patrizia Carlucci, Gloria Pelizzo, Elena Zoia, Giulia Lanfranchi, Silvana Castaldi, Patrizia Boracchi, Elia Biganzoli, Gianvincenzo Zuccotti

**Affiliations:** 1 Department of Internal Medicine, University of Pavia, 27100 Pavia, Italy; 2 Pediatric Department, “V. Buzzi” Children’s Hospital, 20154 Milan, Italy; 3 Medical Statistics Unit, Department of Biomedical and Clinical Sciences, University Hospital, University of Milan, 20157 Milan, Italy; 4 Endocrinology Laboratory Unit, “Luigi Sacco” University Hospital, 20157 Milan, Italy; 5 Intensive Care Unit, “V. Buzzi” Children’s Hospital, 20154 Milan, Italy; 6 Department of Biomedical and Clinical Science, University of Milan, 20157 Milan, Italy; 7 Pediatric Surgery Department, “V. Buzzi” Children’s Hospital, 20154 Milan, Italy; 8 Department of Biomedical Sciences for Health, University of Milan, 20157 Milan, Italy; 9 Fondazione IRCCS Ca’ Granda Ospedale Maggiore Policlinico, 20122 Milan, Italy

**Keywords:** thyroid function, thyroid hormones, disability, neuromotor impairment, metabolic syndrome, children

## Abstract

Thyroid function plays a crucial role in nervous system integrity and metabolic homeostasis. We evaluated the pattern of TSH, FT4 and FT3 release in children with neuromotor impairment (NI) in relationship with metabolic syndrome (MS). We enrolled 55 patients with NI and 30 controls. Clinical parameters, thyroid function and MS presence were recorded. Principal component analysis (PCA), cluster analysis, and logistic regression models were performed. MS was detected in 54.5% of patients. Four clusters were identified: the first one included only controls and, contrasting with cluster 4, was exclusively characterized by children with disability and MS. This latter showed increased FT4 and FT3 and decreased TSH levels. Cluster 2, characterized by disability without MS showed high FT4 and FT3, whereas cluster 3 with low FT4 and FT3 mainly included disability (90%) and showed prevalent MS (57%). The association between TSH and NI is represented by a U-shape structure. The TSH, FT3 and FT4 release patterns may reflect thyrotropic adaptation, allostatic response and compensatory mechanisms. These mechanisms, found in both MS and disability, show that the odds of having a condition of NI with or without MS increase as the TSH values deviate, in both directions, from a value of 2.5 mLU/mL.

## 1. Introduction

Thyroid hormones (THs) play a crucial role in growth, metabolic homeostasis brain development and function [1]. Throughout life, the metabolic pathways are needful to the integrity of various neural processes and an early life exposure to metabolic insults may disturb the proper formation of neurological circuits, leading to neurological impairment and/or neurodegeneration [2].

The thyroid function is involved in different metabolic pathways and THs show multiple effects on glucose and lipid metabolism and blood pressure regulation [3,4,5], influencing the prevalence of metabolic syndrome (MS) [6], which represents a strong predictor of type 2 diabetes, cardio-vascular disease and neurodegenerative diseases [7,8].

Recent data have shown that different patterns of release of THs and thyroid stimulating hormone (TSH), identified for hormones values falling within reference ranges (RR), may be associated with obesity, MS, prediabetes, and diabetes. Although hypothyroidism is the main thyroid disorder, a thyroid profile consistent with both free T4 (FT4) and TSH increase has been detected in early stages and incident diabetes [9]. Furthermore, it has been observed that a gradient of increasing TH levels are still within a normal RR, across the early stages of diabetes [10] and higher odds of insulin resistance and cardiometabolic risk factors have been reported in youth with TSH levels >75th percentiles [11]. However, high TSH levels, which also produce results within normal levels, are associated with increased adiposity, consistent with near-hypothyroidism profiling and with a decreased THs release [12].

In this study, we evaluated the patterns of release of TSH and TH in a population of children with neuromotor disability and its relationship with the presence of MS, compared to a pediatric control group, to define if and how an alteration to and a modulation of thyroid function may have an effect on early metabolic derangement and neuromotor impairment. Moreover, considering the important role of THs in survival and neuronal differentiation in adult age, an early detection of new interrelated hormonal and metabolic parameters may be important for the tailored care and therapy of pediatric patients with a neuromotor disability.

## 2. Patients and Methods

### 2.1. Patients

We enrolled 55 pediatric patients (36 males/19 females, aged 9 years (4, 16 yrs 1° and 3° quartile, respectively) with neuromotor impairment (Level 4–5 according to Gross Motor Function Classification System referred at evaluation)). Diagnoses included cerebral damage in 38 patients (69.1%) (11 hypoxic ischemic damage; 19 dysmorphic syndromes; 8 epileptic encephalopathy) and neuromuscular diseases in 17 (30.9%) (15 spinal muscular atrophy 1–2; 2 Steinert myotonic dystrophy). The patients were referred for endo-metabolic evaluation and/or management of ventilatory support. Clinical parameters, thyroid status and metabolic assessments were recorded for all patients. Goiter was excluded in all enrolled patients. In all subjects with cerebral damage, at least two anticonvulsive drugs were administered. Hormone therapy (including thyroid drugs) was not assumed. Enteral feeding was adopted in 34/55 (61.8%) children (55.8% bolus or 44.2% continuous). No patients live in iodine-deficient areas. As recommended by the national iodine prophylaxis program, iodine sufficiency is maintained through iodine salt fortification. No patients with pathological puberty (precocious or delayed) were included.

Thirty healthy subjects with normal neuro-motor development, comparable for age and sex, referred by their general practitioner or primary care pediatrician for auxological evaluation, were included as controls; the subjects with history of prematurity, low weight at birth (intrauterine growth retardation and/or small for gestational age), documented iodine deficiency and/or thyroid disorders and/or goiter at clinical evaluation were excluded.

### 2.2. Methods

#### 2.2.1. Clinical Examination

In all participants, height (Ht), weight, pubertal stage, waist circumference, WC/Ht, body mass index (BMI), systolic (SBP) and diastolic (DBP) blood pressure measurements were collected.

Height, weight, pubertal stage, WC measurement, DBP and SBP were performed, as reported elsewhere [13]. Pubertal stages were classified as follows, Prepubertal stage 1 = Tanner 1; Middle puberty stage 2 = Tanner 2–3; Late puberty stage 3 = Tanner 4–5 [14].

BMI was calculated as body weight (kilograms) divided by height (meters squared) and was transformed into BMI z scores using WHO reference values [15].

#### 2.2.2. Biochemical Evaluation

All subjects underwent a blood draw in a fasting state between 8:30 a.m. and 9:00 a.m. and plasma glucose, insulin, triglycerides (TG), total and HDL cholesterol levels were evaluated. As a surrogate of insulin resistance (IR), we considered:

- Homeostasis model analysis—insulin resistance (HOMA-IR), calculated as insulin resistance  =  (insulin × glucose)/22.5 [16];
- TyG-index calculated as ln (fasting triglycerides (mg/dL) × fasting plasma glucose (mg/dL)/2) [17].

As previously reported, MS was defined as the presence of at least three of the following risk factors: BMI z score ≤ −2 or ≥2 (we also considered BMI z score ≤ −2 because undernutrition is a risk factor for MS in neurologically impaired children [18]), SBP ≥ 130 mmHg and/or DBP ≥ 85 mmHg; glycemia ≥ 100 mg/dL and/or HOMA-IR ≥ 2.5 if prepubertal stage 1 o ≥ 4 if pubertal stage 2, 3 [19] cholesterol-HDL < 40 mg/dL in females and <50 mg/dL in males; triglycerides ≥ 100 mg/dL (<10 years) or ≥130 mg/dL (≥10 years).

Thyroid profile included: free T3 (FT3), free T4 (FT4) and TSH using the chemiluminescence immunoassay by Alinity/Abbott system (normal ranges: FT3 3.5–6.3 pmol/L; FT4 9–19.3 pmol/L and TSH 0.5–4.2 IU/mL).

#### 2.2.3. Statistical Analysis

All statistical analyses were carried out with R software (version 4.1.2). R Core Team (2022). R: A language and environment for statistical computing. R Foundation for Statistical Computing, Vienna, Austria.

The distributions of the continuous variables were reported as median, first and third quartiles, after performing graphical checks (quantile-quantile plot) on the normality of them. Categorical variables were reported as frequencies and percentages.

For an initial comparison between control children, children with disabilities and children with disabilities and MS, violin plots were made using a kernel density estimator starting from the individual values of the variables. This approach was very useful for detecting distribution patterns that could not be extrapolated using simple box plots. A further step was to construct a scatterplot matrix to see how the continuous variables were correlated with each other across the whole sample of children. For some pairs of variables we found some non-linearities, but, nevertheless, monotonic, spearman coefficients were calculated.

Since pairwise correlations do not consider the joint correlation structure, for a more in-depth study of it we carried out a principal components analysis. This method allowed us to reduce the dimensionality of the dataset by projecting each data point onto the first principal components (up to four) while preserving as much data variation as possible. As some non-linearities were present between the variables, before proceeding further with the multivariate analysis, we first used a more robust approach to assessing the correlation structure, calculating principal components on the ranks of the variables and not on the scaled values. As with the results of the rank, principal component analysis (PCA) confirmed those of the classical approach, and since non-linear components were negligible compared to linear components of the variables, we decided to proceed with the analysis of the results of the classical PCA.

After thoroughly analyzing the association structure of the variables by means of the loading plots, considering the coordinates of the individuals calculated from the principal components, we projected the individuals onto the new planes defined by the first four principal components and labelled the subjects differently with respect to the three study conditions, to understand which dimensions separated the three groups the most. By considering the individual coordinates of the subjects retrieved from PCA, we performed a hierarchical clustering algorithm on principal components to extract some profiles that could be interesting for describing the correlation structure of the data. The cluster obtained by the procedure were then projected onto the planes defined by the first three principal components, as our interest was to characterize them depending on their features synthetized by the PCs. Then, we cross-tabulated the subjects for their membership to one of the three conditions studied (control children, children with disability, children with disability and MS) and their membership to one of the four clusters retrieved from the analysis.

Lastly, since we were specifically interested in studying the relationship between the MS condition, the disability condition, and the thyroid hormonal profiles, we carried out a multinomial logistic regression model, using the three conditions studied in the previous analyses as response variables (being a control child, being a child with disability and being a child with disability and MS). Firstly, we built a different univariable model containing, as a predictor, each single variable related to thyroid hormones. Non-linear effects were considered by modelling restricted cubic splines with three nodes fixed. Only TSH and the non-linear effect of TSH were found to be significantly associated with the response variable studied. Then, we proceeded to build a multivariable multinomial logistic regression model containing, as a predictor, the non-linear effect for TSH adjusted for FT3 and FT4 variables, to model how the odds ratios between a condition of being a child with disability, compared to a condition of being a control child (reference condition in the model), with the change depending on increasing values of TSH. This was also done comparing the odds ratio between a condition of being a child with disability and a condition of being a control child. The non-linear effect was represented by means of scatterplot, representing the log of the odds in the function of increasing values of TSH. A total of 95% C.Is of the effect were computed from the model and represented as dashed lines in the graphs. Finally, to summarize how TSH increases of 0.25 mLU/L are associated with the condition of being a child with disability and with disability and MS, we made a forest plot, where the odds ratios for different ranges of TSH increase for both models were reported.

## 3. Results

In Table 1, metabolic parameters, THs for children with neuromotor impairment and controls are reported.

MS was detected in 30/55 (54.5%) of patients with neuromotor disability. Major differences in metabolic parameters and hormones are evident for children with disability and MS, compared to controls and patients without metabolic derangement. Significant difference in metabolic parameters and hormone levels were shown in children with cerebral vs. neuromuscular diseases Table 2.

**Figure A1:**
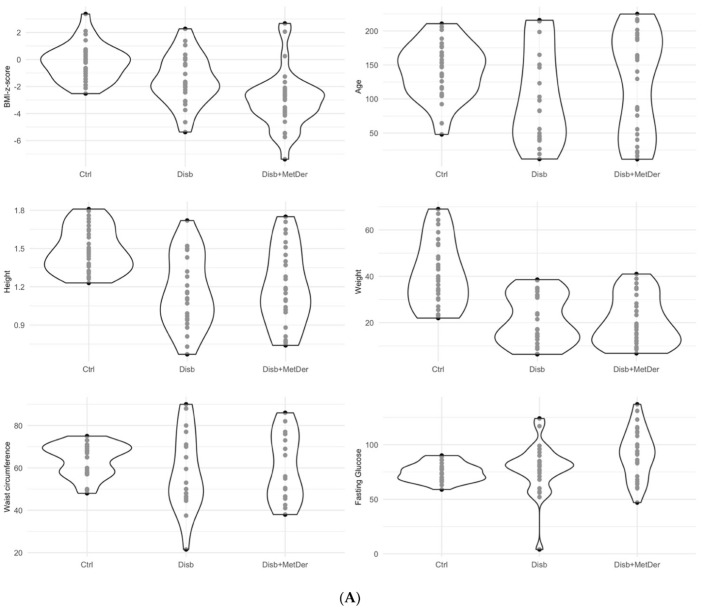

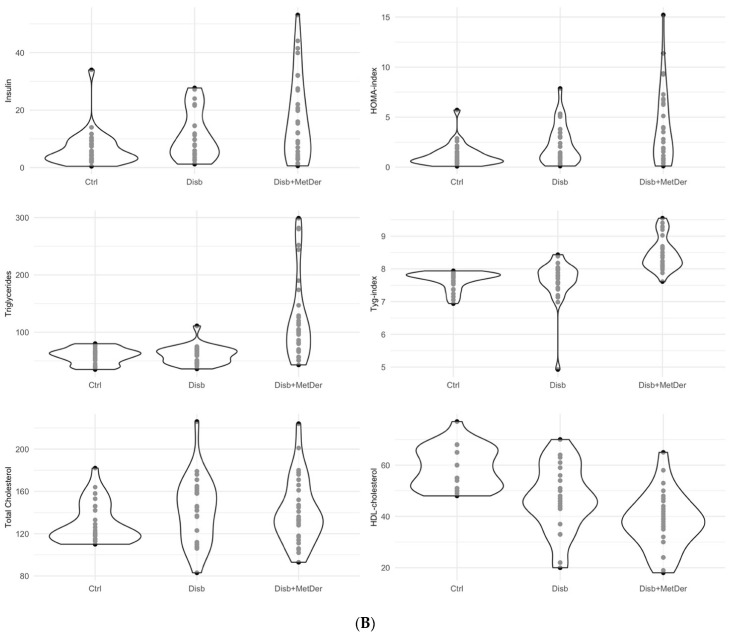

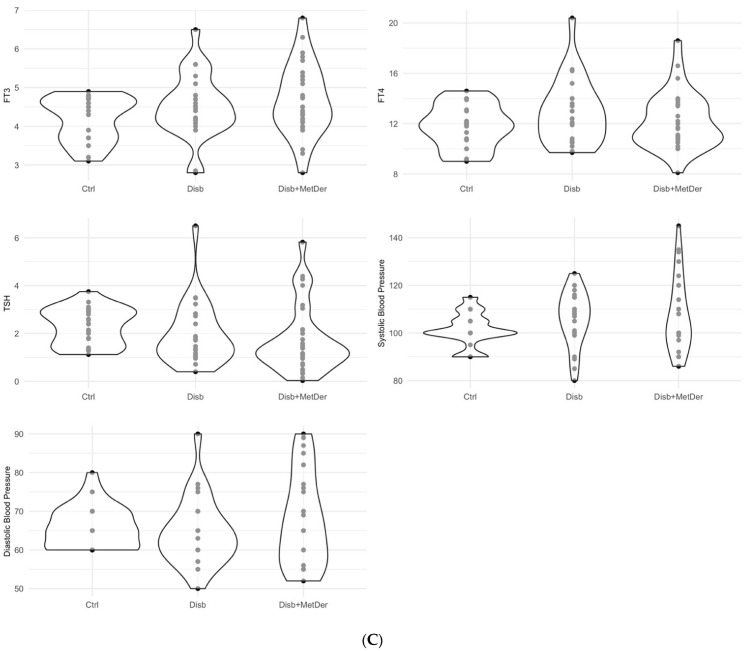
Violin plots showing the distribution of the numeric variables separated for the classes of the group studied. The graphs were distributed in three different panels (**A**–**C**) due to space constraints: Control children (Ctrl), Children with neuromotor impairment (Disb), Children with neuromotor impairment and Metabolic derangement (Disb + MetDer).

The violin plots showing the distribution of the numeric variables separated for the classes of the group studied can be seen in Appendix A, Figure A1, panels a–c.

### 3.1. Principal Component Analysis

Given the correlations between the original variables and the indices derived by them, we decided to synthesize the variables blood glucose and triglycerides with the variable TyG-index; cholesterol total and HDL variables with the fraction of cholesterol-total over cholesterol-HDL variable; and height, age, sex, and weight with the variable BMI-z-score. The variable insulin was considered in the original measurement scale. Systolic and diastolic blood pressure were not included because they were poorly correlated with the other variables. The variables FT3, FT4 and TSH were also considered in the original measurement scale because they were not used in the classification of the three subject groups (control, disability and disability with MS). PCA gave the following results. The cumulative variance explained by the first four principal components is 76% (PC1 = 29%, PC2 = 20%, PC3 = 14%, PC4 = 13%). Since these explained a large part of the variance present in the dataset, we considered them sufficient for the exploration of the association structure of the variables.

**Figure 1:**
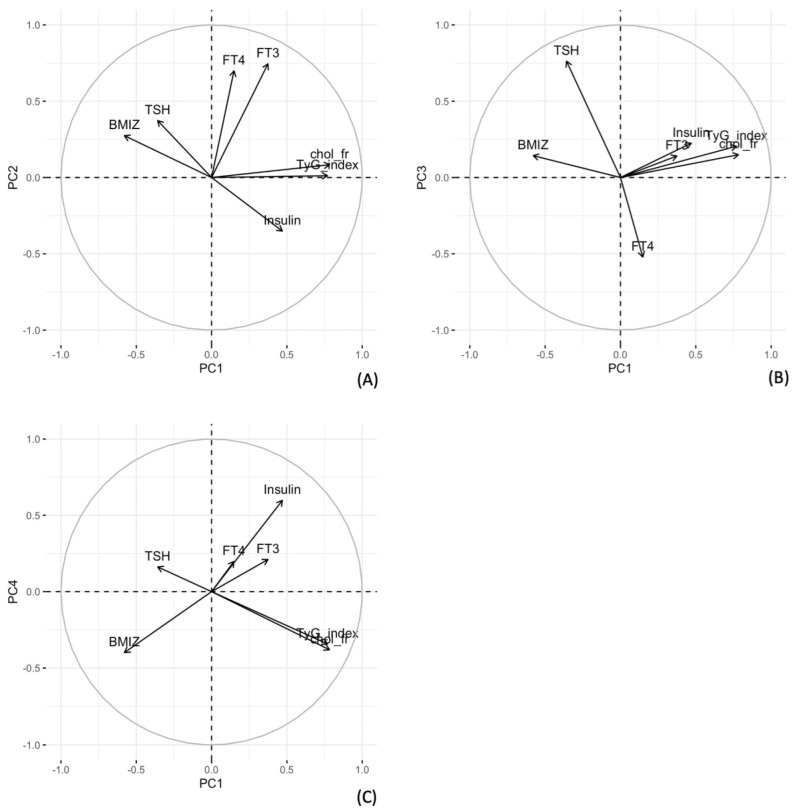
The three subfigures in this panel show the correlation structure studied by PCA. When a significant amount of variability in the data are explained by the first principal components, to get a complete view of the joint relationship between the variables, it is useful to project the vector of variables into a plane defined by two of the PCs. The longer and closer the vector is to the circle, the better a variable is represented by the plane studied. Positive correlation between variables and dimensions occurs when the vectors go in the same direction. Conversely, negative correlation occurs when the vectors of the variables go in the opposite direction. The closer the vectors of the variables are, the greater the correlation between them. In (**A**), the variables are projected in the plane defined by PC1 and PC2. FT4 and FT3 are positively correlated with each other and with PC2, while the ratio of total cholesterol to HDL-cholesterol (chol_fr) and the triglyceride and glucose index (TyG index) are positively correlated with each other and negatively correlated with PC1. The BMI-z-score, TSH and insulin do not seem to be well represented by the plan, as their vectors are short. In (**B**), the TSH seems to be positively correlated with PC3, while in (**B**) the TyG-index and col_fr are positively correlated with each other and negatively correlated with PC1. In (**C**), the BMI-z-score seems to be positively associated with PC1 when it defines the plane with PC4.

In the next step, the loading of each variable to the definition of each specific principal component was calculated and displayed in three graphs of the projection of the variables vectors in the plane defined by the first four principal components (Figure 1).

From the graphs, metabolism-related variables as the cholesterol fraction HDL/Total, TyG index and insulin are positively correlated with PC1, whereas BMI-z-score and TSH is negatively correlated with PC1 (A–C). Moreover, while the variables FT3 and FT4 are positively correlated to PC2 (A), the variable TSH and partly FT4 contribute the most to PC3 and (B), TSH is positively correlated to PC3; FT4, instead, negatively correlated to it. Insulin provided the maximal contribution on PC4 with a positive correlation with this axis.

**Figure A2:**
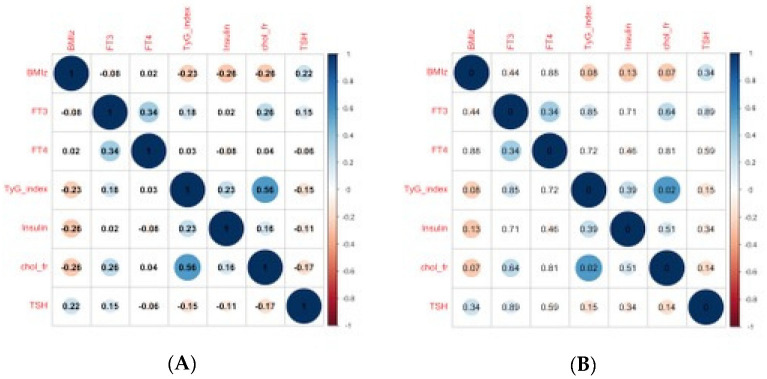
Correlation coefficients (**A**) and *p*-values (**B**) for the associations in PCA analysis.

In Appendix A, Figure A2, the correlation coefficients and *p*-values for the mentioned associations in PCA analysis are shown.

### 3.2. Hierarchical Clustering on Principal Components

**Figure 2:**
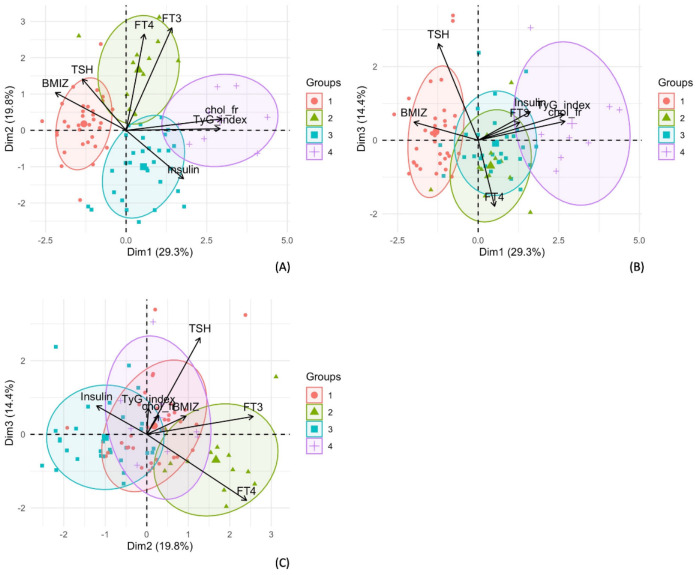
In this panel, the three subfigures show the profiles of each cluster found with HC. These are projected in the planes defined by the first three principal components. In (**A**) cluster one (red), is characterized by high values of PC1, namely, high values of BMI-z-score and high values of TSH. This is opposed to cluster four (purple) that is characterized by the lowest value of PC1, so by high values of total cholesterol to HDL cholesterol ratio (chol_fr) and triglycerides to glucose index (TyG-index). Moreover, cluster two is characterized by high values of PC2, namely, higher values of FT4 and FT3, while cluster three is opposed to two as is characterized by low values of PC2. In (**B**) and (**C**) the separation of the cluster by PC1 and PC2 is shown. Cluster one and four are distinguished by PC1, whereas cluster two and three are distinguished by PC2.

From the hierarchical clustering analysis performed on the individual coordinates retrieved from the first four principal components, a total of four clusters were identified as the optimal number in the dataset. To characterize the clusters, we projected the 85 children observations in the planes defined by PC1–PC2 and PC1–PC3 labeled by the membership of the specific cluster. Additionally, the variable projections were reported as vectors in the same graph. Cluster four is the one characterized by high values of PC1, and thus high values of insulin, TyG index and cholesterol-HDL vs. cholesterol-total ratio. In contrast, cluster one is characterized by low values of PC1, and thus low values of insulin, TyG index, cholesterol total to cholesterol HDL ratio and high values of BMI-z-score (Figure 2).

In addition, cluster two and three oppose each other on PC2, characterizing the hormones FT4 and FT3 (A, C). Specifically, cluster two is characterized by high values of PC2, and thus high values of FT4 and FT3, while cluster three is characterized by low values of PC2, and thus low values of FT4 and FT3.

Considering the cross tabulation of the clusters with the group classification of subjects in the study, we found consistent results. In fact, cluster one is mainly populated by control children (27/34); after all, it was the one characterized by low PC1 values. Instead, clusters two and cluster three are populated mostly by children with disabilities (13/13 and 27/30) but with a substantial difference: while cluster two contains mainly only children with disability (9/13), cluster three contains a remarkably high number of children with disability and MS (17/30).

Cluster four, on the other hand, identifies a condition characterized by high values for MS parameters; in fact, all eight subjects (8/8) in that cluster are with disability and MS.

For a faster comparison and description of the clusters obtained, the distribution of each variable according to cluster membership is reported in Table 3.

**Figure A3:**
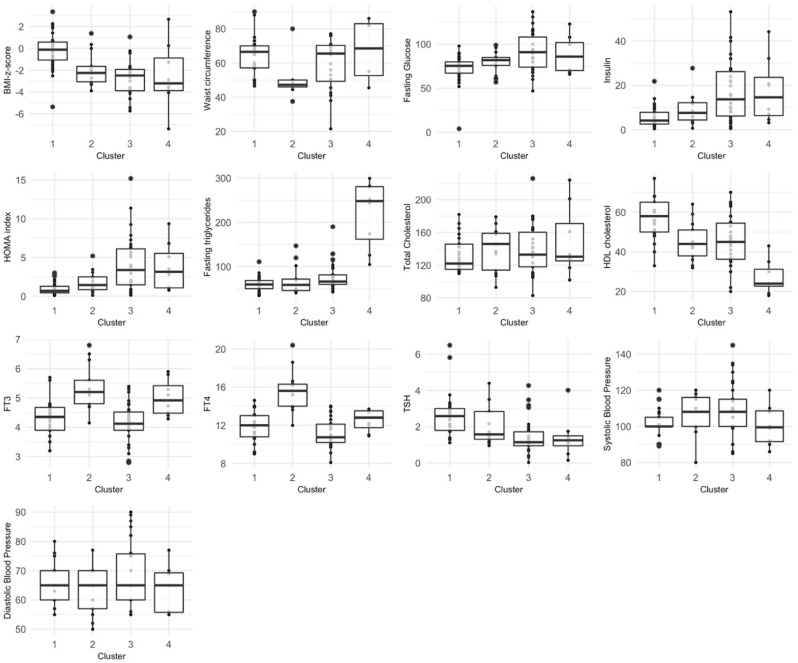
Boxplot showing the distribution of the numeric variables separated by cluster found with hierarchical clustering on principal components.

Regarding the thyroid hormonal profile, cluster two is characterized by lower TSH values than the overall mean of TSH in the studied sample and by lower TSH values than cluster one (control children). When looking at the distribution of FT4 and FT3, it is evident that cluster two has substantially higher values than cluster one. Cluster three is peculiar in that it is characterized by low values of TSH, and thus also of FT4 and FT3 (all of these below the general mean of their respective distributions). Cluster four, characterized by levels of FT3 above the overall mean of FT3 and FT4 has even lower TSH levels than the other clusters. The boxplot showing the distribution of the numeric variables separated by cluster can be seen in Appendix A, Figure A3.

### 3.3. Logistic Regression Models

**Figure A4:**
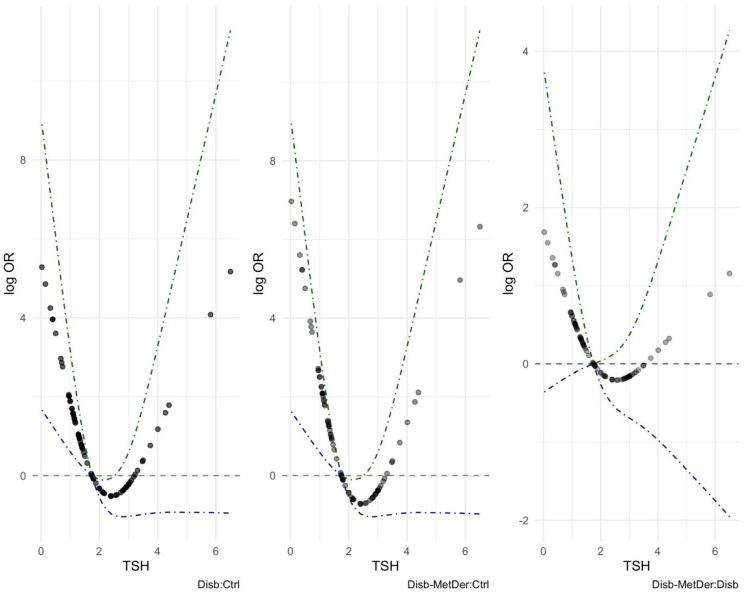
The figure shows the U-shaped statistically significant relationship between increasing values of TSH and the logarithmic form of the odds between the studied conditions found in the univariable multinomial logistic regression model.

In the univariable analysis for FT3, FT4 and TSH, the relationship with the classes of the studied group was analyzed considering both linear and non-linear terms. Regarding FT3 and FT4, the contribution of the non-linear terms was not statistically significant in the models. Conversely, a U-shaped statistically significant relationship was found for TSH (Appendix A, Figure A4).

**Figure 3:**
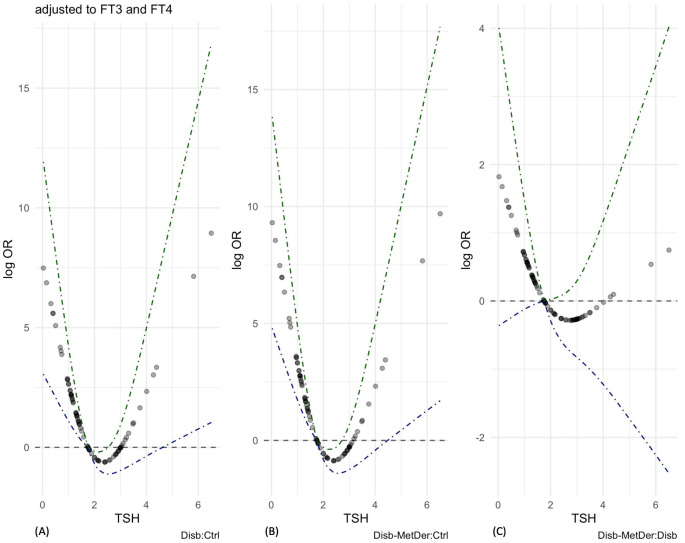
In this panel, the three subfigures represent the non-linear effect of TSH as it is related to the three classes of the groups studied. In all three comparisons (i.e., children with neuromotor impairment versus control children; children with neuromotor impairment and metabolic syndrome versus control children; children with neuromotor impairment and metabolic syndrome versus children with neuromotor impairment only), the probability of being a child with neuromotor impairment with or without MS decreases with increasing TSH levels up to 2.5 mLU/L, then the probability increases with increasing TSH levels above 2.5 mLU/L. Considering the median TSH value (1.75 mLU/L), the confidence intervals of the logarithm of the odds ratios are above the horizontal line set at 0 between the minimum and median TSH value and below between the median value and a TSH value of 2.5 mLU/L. As a function of TSH values above 4.5 mIU/L, they narrow and do not include the horizontal line set at 0 for either of the first two comparisons (**A**,**B**). However, when considering the log odds ratios between children with disabilities and MS and children with disabilities only, although a similar pattern is observed, the confidence intervals are very wide and the horizontal reference line of 0 is always included, with no evidence of association (**C**). Disab = disability; Ctrl = controls; MetDer = metabolic derangement.

In the comprehensive view of the pattern of associations between TSH, TH hormones and disability, a multivariable logistic regression model was fitted. The predictors considered in the model were TSH, with both the linear and non-linear terms, as the contribution of the non-linear terms was statistically significant in the multivariable analysis, and the linear terms only for FT3 and FT4. In addition, since the interactions between TSH and FT3 and TSH and FT4 were not statistically significant, an additive model was considered. The variable coding for the classes of the studied group was set as response variable. When adjusted for FT3 and FT4, the overall pattern of the logarithmic form of the odds ratio between a child with disability and a control child; that of the odds ratio between child with disability and MS and control child; and that of the odds ratio between a child with MS and child with disability displayed a U-shaped relationship with the levels of TSH, as it has also been observed in the univariable analysis (Figure 3).

In fact, the odds to be a child with disability without MS decrease as the TSH levels increase up to 2.5 mLU/L, then the odds increase as the levels of TSH increase above 2.5 mLU/L. Considering the median value of TSH (1.75 mLU/L), the confidence intervals of the logarithm of the odds ratios are above the horizontal line set to 0 between the minimum and the median value of TSH and below between the median value and a value of 2.5 mLU/L of TSH. Moreover, the confidence intervals for the logarithmic form of the odds ratio between a child with disability and a control child and a child with disability with MS and a control child, in the function of values of TSH above 4.5 mLU/L, become narrower and they do not include the horizontal line set to 0. The same pattern can be seen when the odds ratios between a condition of disability with MS and a condition of being a control child are considered. However, considering the pattern of log odds ratios between children with a disability and MS and children with a disability only, a similar behavior is observed, but the confidence intervals are very wide and the horizontal 0 reference line is always included, without the evidence of association.

**Figure 4:**
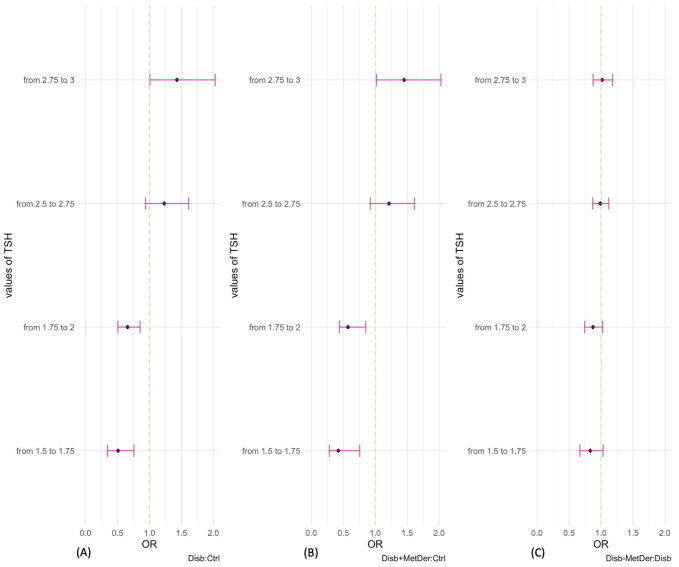
To facilitate the quantitative interpretation of the associations, odds ratios calculated for a fixed increase of 0.25 mLU/L from TSH values of 1.5 mLU/L up to 3 mLU/L are represented in this panel. Considering the association between being a child with neuromotor impairment and a control child and TSH (**A**), the odds ratios ranged from 0.51 to 1.43. Only the increases from 1.5 mLU/L to 1.75 mLU/L and from 1.75 mLU/L to 2 mLU/L are statistically significant. Considering the association between being a disabled child with MS and a control child. (**B**), the odds ratios ranged from 0.42 to 1.45. Only the increases from 1.5 mLU/L to 1.75 mLU/L and from 1.75 mlUL to 2 mLU/L are statistically significant. Considering the association between being a child with a disability and MS and a child with a disability (**C**), the odds ratios range from 0.8 to 1.01.

Given the non-linear pattern of association of TSH, to facilitate the quantitative interpretation of the associations, the odds ratios for a fixed increase of 0.25 mLU/L starting from TSH values of 1.5 mLU/L up to 3 mLU/L were also computed and reported in the forest plot (Figure 4).

Considering the association between being a child with disability and a control child with TSH, the odds ratios range from 0.51 to 1.43. Only the increments from 1.5 mlUL to 1.75 mLU/L and from 1.75 mLU/L to 2 mLU/L are statistically significant. Considering the association between being a child with disability with MS and a control child with TSH, the odds ratios range from 0.42 to 1.45. Only the increments from 1.5 mLU/L to 1.75 mLU/L and from 1.75 mLU/L to 2 mLU/L are statistically significant. Considering the association between being a child with disability and MS and a child with disability with TSH, the odds ratios range from 0.8 to 1.01.

**Figure 5:**
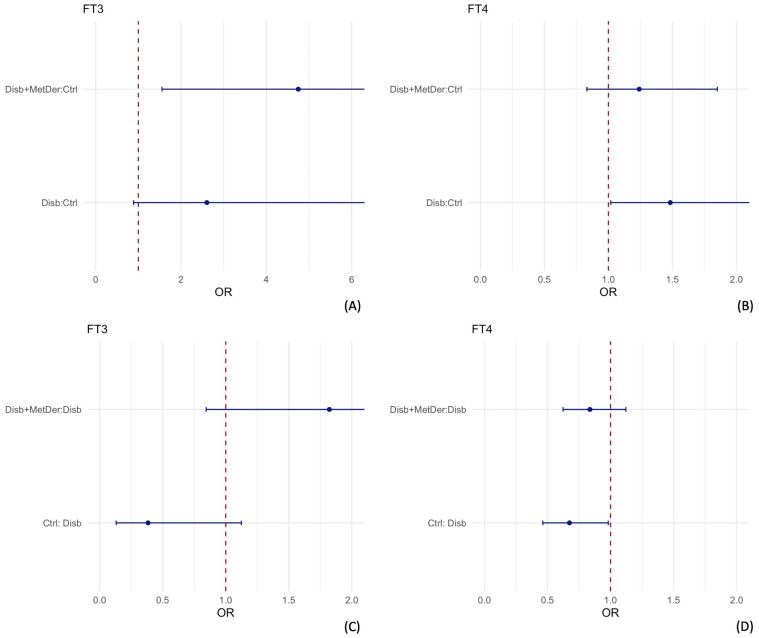
This figure shows the effects of FT3 and FT4 in relation to the classes of the groups studied when adjusted for the other variables included in the model. With a 1 pmol/L increase in FT3, the odds of being a child with or without MS increase, compared to control children (**A**). The same effect occurs with a 1 pmol/L increase in FT4: the odds of being a child with disability with or without MS increase, compared to control children (**B**). When children with a disability are considered as the reference (**C**), for a 1 pmol/L increase in FT3, the odds of being a child with disability and MS increase, compared to children with a disability alone, although there is no statistical evidence of the effect, as the upper confidence limit intersects the vertical line with the x-intercept set at 1. However, for a 1 pmol/L increase in FT4, the odds of being a child with a disability and MS decrease, compared to children with a disability alone, although there is no statistical evidence of the effect, as the upper confidence limit intersects the vertical line with the x-intercept set at 1 (**D**). Disab = disability; Ctrl = controls; MetDer = metabolic derangement.

Regarding the association between FT3 and FT4 and the classes of the studied group, the multivariable model showed that when adjusted for the other variables, with an increase of 1 pmol/L of FT3, the odds of being a child with a disability with or without MS increase respect to control children. The same effect is shown with an increase of 1 pmol/L of FT4: the odds of being a child with disability with or without MS increase, with respect to control children. To evaluate if the presence of MS, in addition to disability, was associated with the levels of THs, the children with disability were considered as a reference group, and we show that for an increase of 1 pmol/L of FT4, the odds of being a child with a disability and MS decrease, with respect to children with a disability only (Figure 5), even though there is no statistical evidence of the effect, as the upper confidence limit intersects value 1.

**Figure A5:**
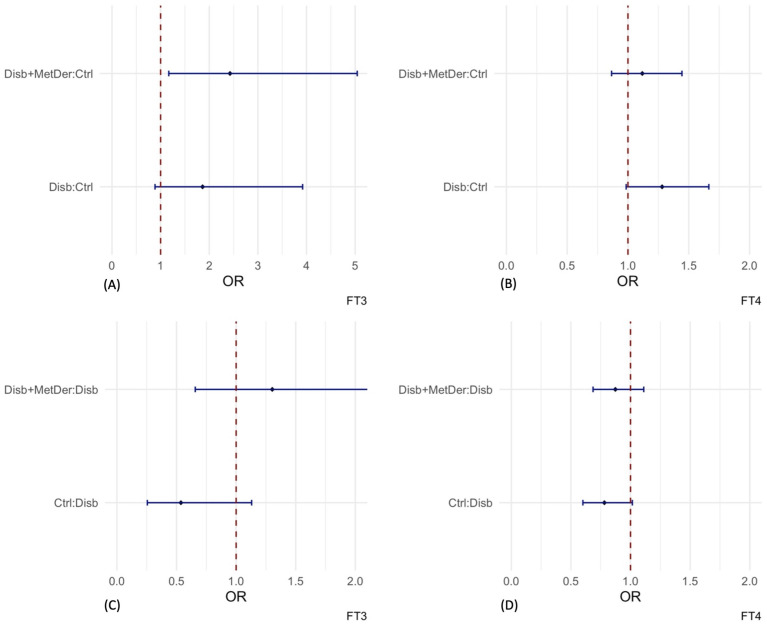
The figure shows the effects of FT3 and FT4 by means of the odds ratio in relation to the classes of the groups studied, when studied in the univariable multinomial logistic regression model. In (**A**), the odds ratio of being a child with disabilities and with metabolic syndrome (MS) vs. a control and the odds of being a child with disabilities without metabolic syndrome (MS) vs. a control child for an increase of 1 pmol/L of FT3; in (**B**), the odds ratio of being a child with disabilities vs. a control child and the odds of being a child with disabilities without metabolic syndrome (MS) vs. a control child for an increase of 1 pmol/L of FT4. In (**C**), the odds ratio of being a child with disabilities and MS vs. a child with neuromotor impairment and without MS, for an increase of 1 pmol/L of FT3, whereas in (**D**), the odds ratio of being a child with disabilities and MS child with neuromotor impairment without MS for an increase of 1 pmol/L of FT4.

The same pattern of association was found in the univariable analysis, reported in Appendix A, Figure A5.

## 4. Discussion

Several pieces of clinical evidence have shown that altered thyroid function plays a key role in the onset and development of various diseases, having a relevant impact on the health-care system, such as MS, diabetes, and cardiovascular disease [1,2,3,4,5]. Furthermore, in recent decades, experimental data on the effects of THs deficiency on the peripheral nervous system (PNS) have fueled the hypothesis that THs release and activity are crucial in normal peripheral nerves development, based on evidence that developmental myelination is a THs dependent process [20,21,22].

In our pediatric case series, PCA followed by cluster analysis allowed us to identify four clusters, in agreement with the clinical characterization of the metabolic profile of the sample studied and to reveal the relationship with TH function tests. Cluster one, associated with the control condition of children, contrasts with cluster four, which is exclusively characterized by children with neuromotor disability and MS with patterns related to their metabolic/lipidic impairment. This latter shows decreased levels of TSH and increased levels of FT4 and FT3. Clusters two and cluster three are separated according to FT4, FT3 levels and insulin. Namely, cluster two, with high FT4 and FT3, is mainly composed by children with disability without MS, whereas cluster three, with low FT4 and FT3, is associated with higher values of insulin and MS condition.

The evidence that cluster two, characterized by increased levels of FT3 and FT4, exclusively constitutes of children with disability, prevalently without MS, may indicate that in these cases a compensatory mechanism might counterbalance the TH deficiency, previously described in the cluster three and/or the lowest peripheral TH activity or bioavailability, hypothesized by the experimental evidence [23]. In cluster three, characterized by high MS prevalence, low levels of THs might be considered to contribute to metabolic derangement. The THs pattern of the cluster four, characterized by patients with disability and MS, has been described by other authors to be associated with obesity, diabetes, MS or phenotypes, usually related to hypothyroidism [23,24]. In our case series, this pattern likely reflects an increased set-point of homeostasis, as observed in type 2 allostasis, that results from an expected increase in energy demand, although the cumulative energy balance is still sufficient [25]. Indeed, we have previously reported that a high allostatic load characterizes neurological impaired subjects and changes in biological set points are functions of chronic stressors [26]. The primary mediators of allostatic response include other hormones of the hypothalamus–pituitary–adrenal (HPA) axis and, noteworthily, a complex interplay between HPA hormones and metabolism has been recently described [26,27,28,29].

Undoubtedly in the current literature there are several controversial data on the association between MS and TSH and/or THs, since non-linear effects require investigation by proper statistical methods and typical U-shaped relationships between hormones and clinical conditions require explanation.

The U-shaped relationship between TSH levels and risk of disability substantially may be explained by two physio-pathological mechanisms: the first characterized by lower and the second by higher TSH levels, with respect to 2.5 mLU/mL, although these TSH levels fall within the conventional reference ranges. The pattern characterized by low TSH may be associated with the adaptive response of the HPA axis to allostasis, depending on the severity and duration of the disease. Chatzitomaris et al. [25], have described the long-term adaptation in chronic disease, which results in thyrotropic adaptation characterized by a loss of pulsatility of TSH secretion, a reduced TRH-gene expression and suppressed hypothalamic stimulation. During the normal and vital process of allostasis, the body adjusts its physiological system in response to internal and external stressors. However, constant and/or prolonged stressors create wear and tear on the system, leading to allostasis that is significant contributor to altered hormonal profile and increased morbidity and mortality in persons with disabilities [26]. The pattern characterized by TSH increase could theoretically mirror the experimental condition of hypothyroidism, which has been originally and mainly associated with neuromotor impairment, but the expected TH decrease is counterbalanced in our case series by increased levels of FT4 and FT3. Indeed the model has shown that as FT4 and FT3 increase the odds of being a child with disability with or without MS increases, with respect to the control children. In the literature, a joint increase in TSH and TH has been yet reported for some metabolic diseases traditionally associated with hypothyroidism as diabetes [10]. Conversely, the result that there is no evidence of statistically significant interactions between THs and TSH may reflect a gap in the hypothalamic–pituitary–thyroid feedback loop and the onset of compensatory mechanisms.

This study suffers for some limitations. First, being a pilot study, the sample size may be small and, therefore, might not be representative of the whole population of children with neuromotor disability, which is further characterized by a heterogeneous pathophysiology. Additionally, pubertal hormones might modulate thyroid function and the release of TH; puberty could be considered as a confounding factor in data interpretation. Thus, further investigations, including more patients and collecting data on hormones involved in the HPA axis (gonadotropins, ACTH, adrenal hormones, prolactin), will allow the improvement of this preliminary evidence.

To date, the complex crosstalk between different hormones involved in HPA axis and the resulting effects on thyroidal balance and on metabolism have been widely hypothesized but not clarified at all. Second, we considered only pediatric patients with severe neuromotor impairment, but an extensive evaluation of those with different degrees of neuromuscular damage should be considered to allow a thorough characterization of the role of THs in the ontogenetic development of neuromuscular system and support new therapeutic applications for nerve regeneration. Additionally, to include a reference group with MS and without neuromotor impairment could be useful to understand if the thyroid response is similar or different. Finally, only weight was associated in an opposite fashion of TSH with FT4; in the complexity of clinical condition of these patients, it is not easy to explain it. Height and weight are highly dependent on age during childhood, influencing the correlation between TSH and thyroid hormones. In these children with a disability, the relationship between auxological parameters and thyroid hormones could also be influenced by different physiological timing of puberty and physical inactivity; additionally, the role of inheritable factors could not be excluded.

## 5. Conclusions

Our clinical findings contribute the further evidence that the simple interpretation of TSH and THs levels, according to their conventional reference intervals, cannot capture the patterns of release of the joint hormones, which have recently emerged to characterize MS and diabetes. A joint extensive analysis of TSH and THs release patterns, as the one performed here, is, therefore, required to show how thyroid function ranks at the crossroad of several evolving areas, such as the management of metabolic derangement and the treatment of disability, through peripheral nerves recovery and clinical conditions both characterizing our pediatric sample.

Experimental data have hypothesized THs deficiency and/or an alteration of the permanent expression of TH receptor (TR) in motor and sensory neurons that may affect the development and integrity of neural processes [23,30,31]. Our results have shown that in the clinical setting of disability, a decreased pattern of TSH release might be the initial key of neurological impairment/neurodegeneration.

Indeed, in these children, the pattern of release of THs may be decreased or increased, and in the former case we may hypothesize that a basic TH deficiency tends to be counterbalanced by a compensatory mechanism, which, however, is not enough to revert impaired Schwann cell proliferation/deregulation and to promote fiber regeneration and myelination [22,32]. Noteworthily, there is evidence that only the exogenous administration 3,3′,5-triiodothyronine (T_3_) stimulates the regeneration of severed nerves with the recovery of sensory conduction [23,30,31].

Further studies on the development of reliable algorithms that support the interpretation of thyroid homeostasis in the clinical setting may be helpful for future thyroid research by considering that TSH and THs play a key role on the development of several diseases having a relevant impact on the health-care systems.

## Figures and Tables

**Table 1:** Metabolic parameters, thyroid hormones in patients with neuromotor impairment and controls.

	Controls	Patients with Neuromotor Impairment	
		Without Metabolic Syndrome	With Metabolic Syndrome
n	30	25	30
Age, years (median (IQR))	12.30 (9.15, 13.96)	8.15 (3.75, 12.54)	12.39 (4.17, 15.79)
BMI sds z-score, (median (IQR))	−0.17 (−1.18, 0.54)	−1.76 (−2.43, −0.32)	−2.94 (−4.04, −2.30)
Sex, (n (%))	20 M (67%)	16 M (64%)	20 M (67%)
Puberal Stage, (n (%)) 1	10 (33%)	16 (64%)	15 (50%)
2	13 (43%)	4 (16%)	3 (10%)
3	7 (23%)	5 (20%)	12 (40%)
Fasting blood glucose, mg/dL (median (IQR))	74.00 (68.00, 80.00)	80.00 (71.00, 85.00)	92.00 (74.00, 106.00)
Insulin, µU/mL (median (IQR))	1.47 (1.05, 2.20)	2.08 (1.48, 2.68)	2.49 (1.40, 3.24)
Tryglicerides, mg/dL (median, (IQR))	60.50 (51.00, 66.75)	61.00 (47.00, 70.00)	103.00 (68.50, 142.50)
FT3, pmol/L (median (IQR))	4.35 (3.70, 4.68)	4.46 (4.08, 4.80)	4.50 (4.11, 5.27)
FT4, pmol/L (median (IQR))	12.00 (10.72, 13.07)	12.40 (10.60, 14.00)	11.65 (10.72, 13.50)
TSH, mIU/L (median (IQR))	2.40 (1.49, 2.98)	1.72 (1.06, 2.73)	1.27 (0.96, 2.10)
Cholesterol-HDL, mg/dL (median (IQR))	60.00 (51.00, 65.00)	46.00 (43.00, 56.00)	37.50 (30.50, 43.75)
Cholesterol-total, mg/dL (median (IQR))	120.00 (115.00, 145.00)	142.00 (112.00, 161.00)	133.00 (120.50, 158.75)
HOMA-IR (median (IQR))	0.80 (0.47, 1.52)	1.44 (0.84, 3.06)	2.65 (0.86, 6.35)
Tryg-index (median (IQR))	7.76 (7.57, 7.86)	7.77 (7.55, 7.99)	8.38 (8.11, 8.64)
Systolic blood pressure, mmHg (median (IQR))	100.00 (100.00, 105.00)	108.00 (100.00, 115.00)	100.00 (99.00, 118.50)
Diastolic blood pressure, mmHg (median (IQR))	65.00 (60.00, 70.00)	65.00 (60.00, 70.00)	65.00 (56.00, 75.75)

**Table 2:** Metabolic parameters, thyroid hormones in patients with cerebral damage and neuromuscular diseases.

Variables	Cerebral Damage	Neuromuscular Diseases
*n*	38	17
Fasting blood glucose, mg/dL (median (IQR))	85.00 (68.00, 100.00)	85.00 (76.00, 93.00)
Insulin, µU/mL (median (IQR))	2.21 (1.39, 3.09)	2.47 (1.77, 3.00)
Tryglicerides, mg/dL (median, (IQR))	72.00 (56.25, 109.50)	70.00 (47.00, 102.00)
FT3, pmol/L (median (IQR))	4.21 (3.98, 4.70)	5.30 (4.70, 5.60)
FT4, pmol/L (median (IQR))	11.35 (10.50, 13.47)	13.50 (12.00, 15.60)
TSH mUI/L (median (IQR))	1.44 (0.95, 2.97)	1.41 (1.10, 1.74)
Cholesterol-HDL, mg/dL (median (IQR))	40.50 (33.50, 47.00)	44.00 (35.00, 54.00)
Cholesterol-total, mg/dL (median (IQR))	133.00 (114.75, 160.25)	146.00 (133.00, 161.00)
HOMA-IR (median (IQR))	1.67 (0.79, 5.00)	2.50 (1.44, 3.51)
Tryg-index (median (IQR))	8.12 (7.88, 8.43)	7.97 (7.58, 8.18)
Systolic blood pressure, mmHg (median (IQR))	108.00 (99.25, 115.75)	100.00 (100.00, 115.00)
Diastolic blood pressure, mmHg (median (IQR))	65.00 (57.75, 76.00)	65.00 (60.00, 65.00)
status (%)		
0	0 (0.0)	0 (0.0)
1	14 (36.8)	11 (64.7)
2	24 (63.2)	6 (35.3)

**Table 3:** The table describes the composition of the clusters obtained from the HCPC in terms of prevalence of the classes of the groups of children studied. Whereas cluster one mainly identifies the group of control children (in fact, 79% are control children), clusters two, three and four are mainly composed of children with neuromotor impairment (only cluster three contains 10% control children). Whereas cluster four mainly identifies the condition of impairment with metabolic syndrome, clusters two and three are much more mixed. Indeed, cluster two contains mainly children with disabilities only (69%) but is nevertheless characterized by 33% children with neuromotor impairment and metabolic syndrome, whereas cluster three mainly contains children with disabilities and metabolic syndrome (57%) but is nevertheless characterized by 33% children with disabilities only.

Cluster/Conditions	Control	Disability	Disability + Metabolic Derangement	Total
1	79% (27)	18% (6)	3% (1)	100% (34)
2	0% (0)	69% (9)	31% (4)	100% (13)
3	10% (3)	33% (10)	57% (17)	100% (30)
4	0% (0)	0% (0)	100% (8)	100% (8)
Total	35% (30)	29% (25)	35% (30)	100% (85)

## Data Availability

The data presented in this study are available on request from the corresponding author.
